# Medial–lateral posterior tibial slope asymmetry is associated with the presence of medial meniscus posterior root tear

**DOI:** 10.1186/s43019-026-00352-2

**Published:** 2026-10-29

**Authors:** Kento Harada, Yu Mori, Takahisa Abe, Takashi Aki, Masayuki Kamimura, Masanori Ogasawara, Kentaro Ito, Toshimi Aizawa

**Affiliations:** 1 Department of Orthopaedic Surgery, Tohoku University Graduate School of Medicine, Sendai, Japan; 2 Iwate Prefectural Central Hospital, Morioka, Japan; 3 Sendai Orthopaedic Hospital, Sendai, Japan

**Keywords:** Medial meniscus posterior root tear, Posterior tibial slope, Slope asymmetry, Propensity score matching, Magnetic resonance imaging

## Abstract

**Background:**

Posterior tibial slope (PTS) has been identified as an important anatomical risk factor for medial meniscus posterior root tear (MMPRT). However, prior studies have primarily focused on the medial slope. The primary aim of this study was to measure medial and lateral PTS on magnetic resonance imaging (MRI) and to determine whether medial–lateral slope asymmetry is associated with MMPRT.

**Methods:**

This multicenter retrospective study included patients aged 50–75 years who underwent knee MRI for knee pain at two institutions. Patients with osteoarthritis (Kellgren–Lawrence grade 3 or 4) were excluded. MMPRT was diagnosed on MRI. The medial PTS (MPTS), lateral PTS (LPTS), medial proximal tibial angle (MPTA), and lateral distal femoral angle (LDFA) were measured, and ΔPTS was defined as MPTS − LPTS. Groups were compared before and after 1:1 propensity score (PS) matching for age, sex, and body mass index. Multivariable logistic regression and receiver operating characteristic (ROC) curve analysis were conducted to evaluate the association and discriminative ability of ΔPTS.

**Results:**

A total of 120 knees were included, of which 26 (21.7%) demonstrated MMPRT. MPTS did not differ significantly between groups, whereas the MMPRT group showed a significantly smaller LPTS (*p* < 0.001) and larger ΔPTS (*p* < 0.001). These findings remained consistent after 1:1 PS matching (22 matched pairs), with ΔPTS remaining significantly greater in the MMPRT group (*p* < 0.001). In multivariable analysis, ΔPTS was independently associated with MMPRT (odds ratio 2.17 per 1°, 95% CI 1.56–3.02, *p* < 0.001). ROC curve analysis demonstrated good discriminative performance of ΔPTS for identifying MMPRT (area under the curve [AUC] = 0.87, 95% CI 0.79–0.95), which exceeded that of LPTS (0.73) and MPTS (0.54) (DeLong *p* = 0.006 and *p* < 0.001, respectively). The exploratory cutoff value was 4.0°, yielding a sensitivity of 80.8% and a specificity of 81.9%.

**Conclusions:**

Knees with MMPRT exhibited a smaller LPTS and greater ΔPTS compared with knees without MMPRT, which remained significant after PS matching. ROC analysis demonstrated good discriminative performance, and a ΔPTS of 4.0° or greater may serve as an exploratory threshold. PTS asymmetry may represent a morphological factor associated with MMPRT.

Level of evidence III.

**Supplementary Information:**

The online version contains supplementary material available at https://doi.org/10.1186/s43019-026-00352-2.

## Introduction

Medial meniscus posterior root tear (MMPRT) is a condition in which the posterior root of the medial meniscus is disrupted [1]. The posterior root anchors the meniscus and converts axial load into circumferential hoop stress. Its disruption compromises this hoop mechanism, altering knee kinematics and increasing tibiofemoral contact pressure to a level comparable to that of total meniscectomy [2, 3]. Loss of hoop tension also leads to medial meniscal extrusion, which further reduces the load-bearing area and concentrates stress on the articular cartilage [4]. These mechanical alterations accelerate cartilage degeneration and osteoarthritis progression, and nonoperative treatment is associated with worsening arthritis and poor clinical outcomes [5]. Tear morphology further influences clinical outcome, and it has been shown to be an independent determinant of pain relief after arthroscopic partial meniscectomy in middle-aged patients [6]. Therefore, early identification of knees at risk of MMPRT is clinically important.

Multiple risk factors for MMPRT have been proposed, including patient characteristics such as age and body mass index (BMI) as well as anatomical factors [7]. Among the anatomical factors, posterior tibial slope (PTS) has emerged as one of the most consistently reported morphological parameters associated with MMPRTs [7, 8]. In particular, an increased medial PTS has been identified as a major anatomical risk factor for MMPRT, as it induces anterior tibial translation (ATT) and excessive tensile stress on the posterior horn of the medial meniscus, predisposing it to root failure [9]. Several studies have investigated PTS on plain radiographs in relation to MMPRT [7, 10], although most have focused on the medial slope, and the lateral PTS and the medial–lateral slope relationship have received little attention in this context. This gap is potentially important because the sagittal geometry of the two compartments jointly governs tibiofemoral rotational kinematics. A previous study reported that the difference between the medial and lateral posterior tibial and meniscal slopes is associated with altered in vivo tibial rotation [11]. Moreover, medial–lateral slope asymmetry has been identified as a morphological factor in lateral meniscus posterior root tears [12, 13]. Therefore, we hypothesized that the presence of MMPRT is associated not only with the medial PTS but also with the lateral PTS, and that the difference between the two slopes is more informative than either slope alone.

The primary aim of this study was to measure the medial and lateral PTS on magnetic resonance imaging (MRI) and to investigate how the difference between the medial and lateral PTS angles is associated with the presence of MMPRT.

## Methods

### Participants

This multicenter retrospective study included patients aged 50–75 years who underwent knee MRI for the evaluation of knee pain at the two institutions. Patients were enrolled between January 2014 and December 2025 across the two participating institutions. All patients who underwent knee MRI during this period were screened consecutively from the imaging records of each institution, and the exclusion criteria listed below were then applied. Because the cohort was assembled by retrospective review of imaging records, the number of patients excluded by each individual criterion was not recorded. All MRI examinations were performed using either a 1.5-T or 3.0-T Optima MR430s extremity scanner (GE Healthcare, Milwaukee, WI, USA) at institution A and a 1.5-T scanner (MAGNETOM Essenza, Siemens Healthineers, Erlangen, Germany) at institution B.

The exclusion criteria were as follows: (1) patients with symptomatic osteoarthritis defined as Kellgren–Lawrence grade 3 or 4, (2) patients with acute trauma-related knee pain, or a history of lower limb fracture surgery or knee osteotomy, (3) patients with any concomitant knee ligament injury, regardless of severity, including medial collateral ligament, lateral collateral ligament, anterior cruciate ligament, or posterior cruciate ligament injuries, and (4) patients with any condition causing structural joint destruction, including rheumatoid arthritis or other inflammatory arthritis, crystalline arthropathy (gout or pseudogout), septic arthritis, and hemophilic arthropathy.

### Radiographic measurements

**Fig. 1:**
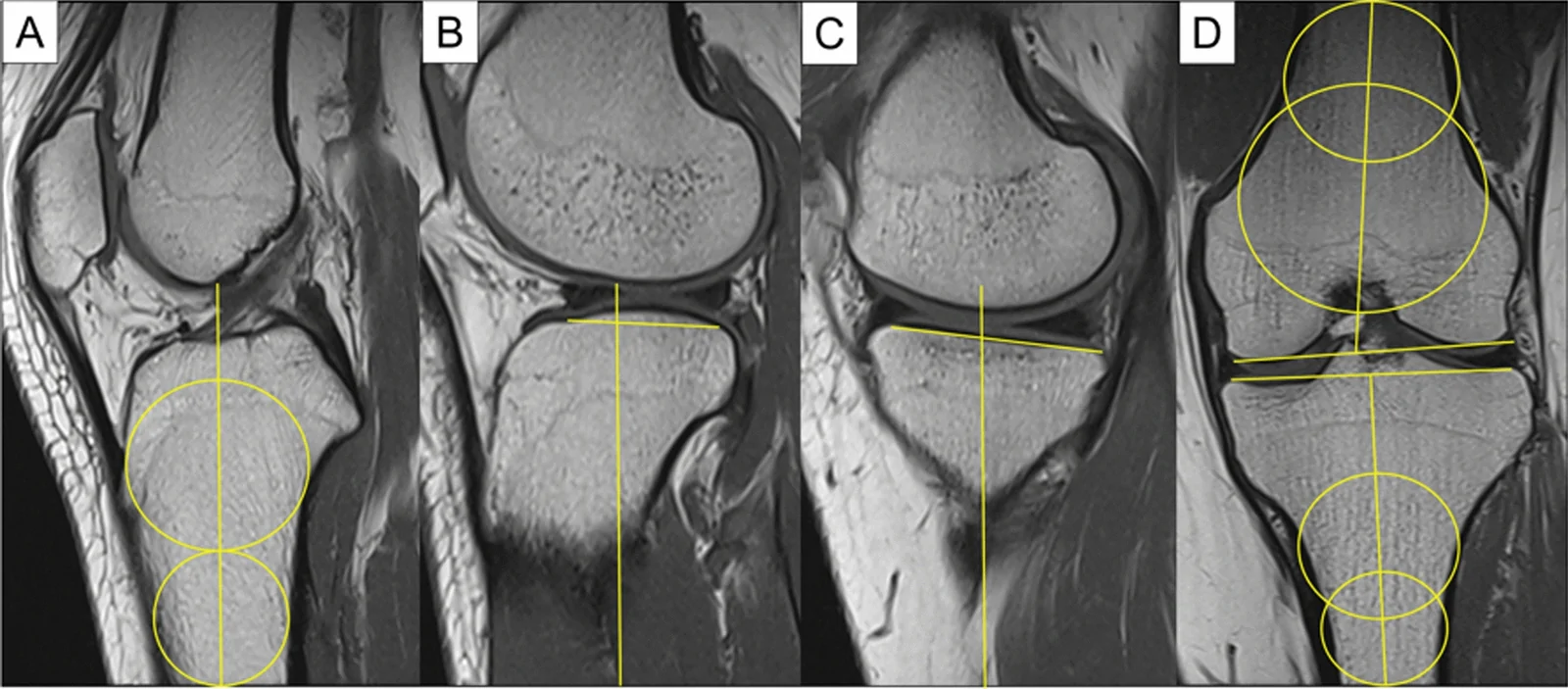
Measurement of posterior tibial slope and coronal alignment on MRI. The tibial longitudinal axis was defined on the central sagittal slice of the proximal tibia using two reference circles (**A**). Sagittal slices corresponding to the mediolateral centers of the medial and lateral tibial plateaus were then selected. Posterior tibial slope was calculated as the angle between a tangent to the subchondral surface and a line perpendicular to the tibial longitudinal axis (**B**, **C**). The medial proximal tibial angle and the lateral distal femoral angle were measured using bone axes constructed with reference circles fitted to the diaphyseal cortices (**D**)

PTS was measured using sagittal MRI according to the method described by Hudek [14] (Fig. 1). First, the central sagittal slice of the proximal tibia was identified as the image that best showed the anterior and posterior cortices and the intercondylar region. On this slice, two reference circles were placed within the proximal tibia to establish a reproducible longitudinal axis. The superior circle was adjusted to contact the anterior, posterior, and superior cortices, whereas the inferior circle was fitted to the anterior and posterior cortices and positioned so that its center lay on the perimeter of the superior circle. A line connecting the centers of these circles was defined as the MRI-based tibial longitudinal axis. Subsequently, separate sagittal slices representing the mediolateral center of the medial and lateral tibial plateaus were selected. On each slice, a tangent was drawn along the subchondral surface by connecting the most superior anterior and posterior cortical edges. The PTS was calculated as the angle formed between this tangent and a line perpendicular to the tibial longitudinal axis. Coronal alignment was assessed on MRI by measuring the medial proximal tibial angle (MPTA) and the lateral distal femoral angle (LDFA) according to the definitions by Paley [15]. The tibial and femoral axes were each defined by two reference circles fitted to the outer cortical margins of the diaphysis, with a line through the circle centers taken as the axis, using a two-circle technique analogous to that used for PTS. Since the femoral mechanical axis cannot be constructed on a knee MRI, the anatomical (bone-axis) LDFA was measured, and the mechanical LDFA was estimated by adding a fixed correction of 6°, corresponding to the mean femoral anatomical–mechanical axis angle [16].

All radiographic measurements were performed using a digital measurement tool in the Picture Archiving and Communication System (PACS): Vue PACS (version 12.2.5.4000286; Carestream Health, Rochester, NY, USA) at institution A and J-Medical 7 (Fuji I.T. Co., Tokyo, Japan) at institution B. Because the measured angles (PTS, MPTA, and LDFA) were each defined relative to a bone axis constructed within the image, they are independent of absolute image scale and field strength, and identical acquisition planes, slice-selection criteria, and measurement protocols were applied to images from both institutions. All measurements were performed by the first author. To assess interobserver reliability, a co-author independently repeated the measurements in a subset of approximately half of the knees. Both observers are board-certified orthopedic surgeons and were blinded to the MMPRT status of each knee and to each other’s values. Interobserver reliability was assessed using intraclass correlation coefficients (ICC [2, 1]) for each measured angle and for ΔPTS.

**Fig. 2:**
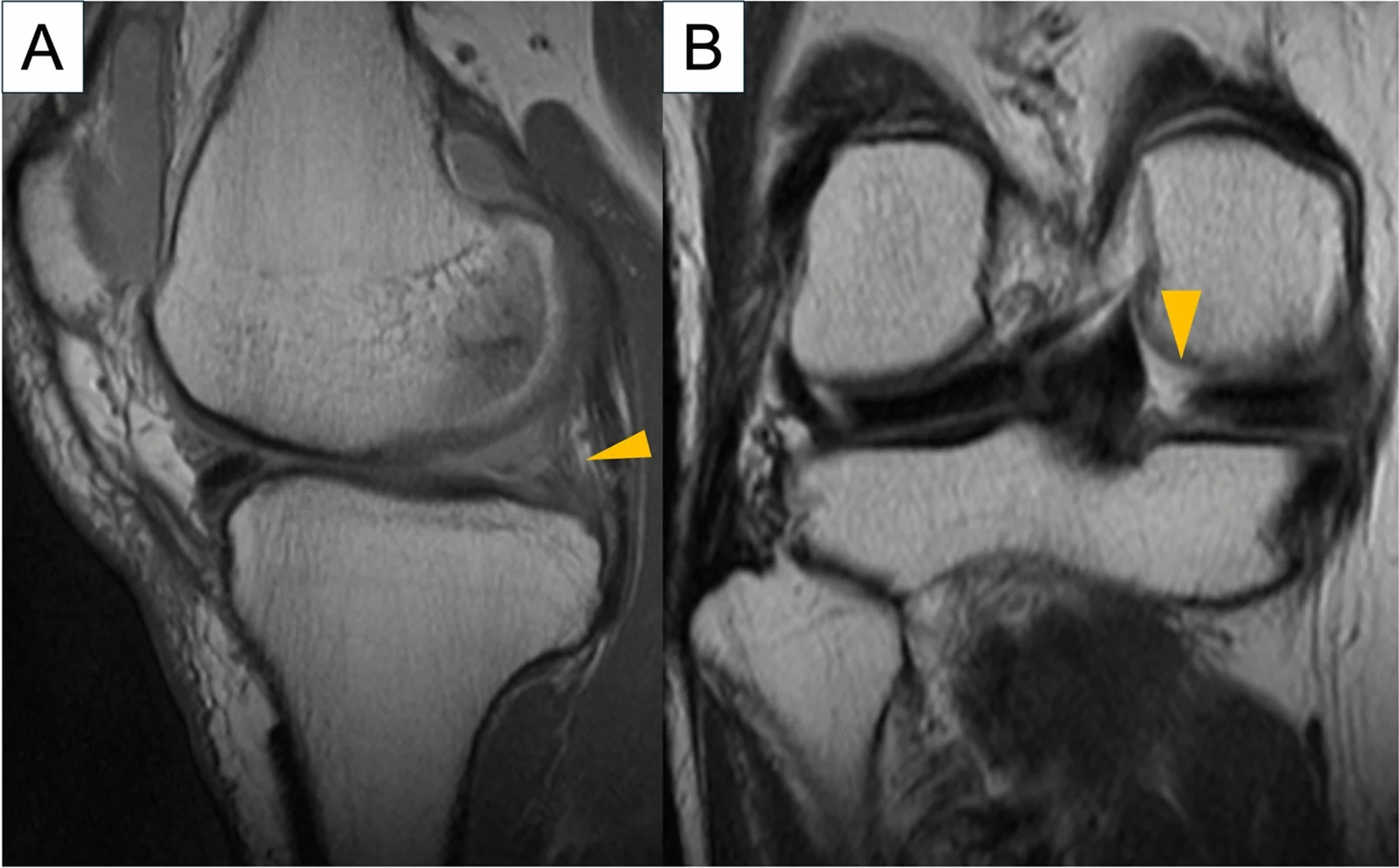
Representative MRI findings used for the diagnosis of medial meniscus posterior root tear (MMPRT). (**A**) Ghost meniscus sign observed on sagittal MRI, characterized by the absence of the normal triangular meniscal signal at the posterior horn (arrowhead). (**B**) Vertical linear defect at the medial meniscus posterior root on coronal MRI (arrowhead)

The presence of MMPRT was determined from MRI by the first author. Knees demonstrating the ghost meniscus sign on MRI [17] or a vertical linear defect [18] were classified as having MMPRT (Fig. 2), and knees without either finding were classified as control. This classification was used for all analyses. To assess its reliability, the same co-author independently reviewed approximately half of the examinations (*n* = 57). This reviewer was blinded to the original classification and to all clinical and measurement data. Agreement between the two readers was quantified using Cohen’s kappa.

### Statistical analysis

We defined the medial PTS as MPTS and the lateral PTS as LPTS. The difference between MPTS and LPTS was defined as ΔPTS (MPTS − LPTS). Baseline characteristics and measurements were first compared between the control and MMPRT groups. Normality was assessed using the Shapiro–Wilk test. All continuous variables were normally distributed and were compared using Student’s *t*-test. Categorical variables were compared using the chi-squared test.

Propensity scores (PS) were estimated by logistic regression with age, sex, and BMI as covariates. Matching was performed by the nearest-neighbor method without replacement, with exact matching on sex and a caliper width of 0.2 standard deviations of the logit of the PS. Within this caliper, the control knee with the smallest Mahalanobis distance for age and BMI was selected [19]. The main analysis used 1:1 matching, and 1:2 matching with the identical algorithm was performed as a sensitivity analysis. Balance was assessed using standardized mean differences (SMDs). After matching, continuous variables were compared using the paired *t*-test in the 1:1 analysis and, because each matched set contained two controls, cluster-robust linear regression with standard errors clustered on the matched set in the 1:2 analysis was performed.

To assess whether ΔPTS was independently associated with MMPRT, multivariable logistic regression was performed in the full cohort with MMPRT as the dependent variable. Covariates were specified in advance on clinical grounds rather than selected based on baseline statistical significance. BMI was included as an established risk factor for MMPRT, and MPTA and LDFA were included as measures of coronal alignment that may influence medial compartment loading. Given the limited number of MMPRT events (*n* = 26), the primary model was deliberately restricted to four covariates to avoid overfitting. We therefore evaluated age and sex in an additional sensitivity model rather than including them in the primary model. ΔPTS, MPTS, and LPTS are exactly linearly dependent and were therefore entered in separate models. A model containing MPTS and LPTS simultaneously was also fitted, and model fit was compared across all models using the Akaike information criterion (AIC), for which lower values indicate better fit. Odds ratios (ORs) with 95% confidence intervals (CIs) were calculated.

Because the study was conducted at two institutions using different MRI systems, patient characteristics and slope measurements were compared between institutions, and the primary comparisons were repeated within each institution. As sensitivity analyses, models further adjusted for age and sex, and for institution, were also fitted.

Receiver operating characteristic (ROC) curve analysis was performed to evaluate the discriminative ability of ΔPTS for identifying the presence of MMPRT. The area under the curve (AUC) was calculated [20], and an exploratory cutoff value was determined based on the Youden index. The AUCs of ΔPTS, MPTS, and LPTS were compared using the DeLong test for correlated ROC curves [21].

Statistical significance was set at *p* < 0.05. The propensity score analyses, the multivariable logistic regression models, and the DeLong test were performed using R version 4.6.1 (R Core Team, R Foundation for Statistical Computing, Vienna, Austria). All other analyses were performed using JMP 18 student edition (SAS Institute Japan, Tokyo, Japan), and graph generation was conducted using GraphPad Prism 10 (GraphPad Software, Boston, MA, USA).

## Results

**Fig. 3:**
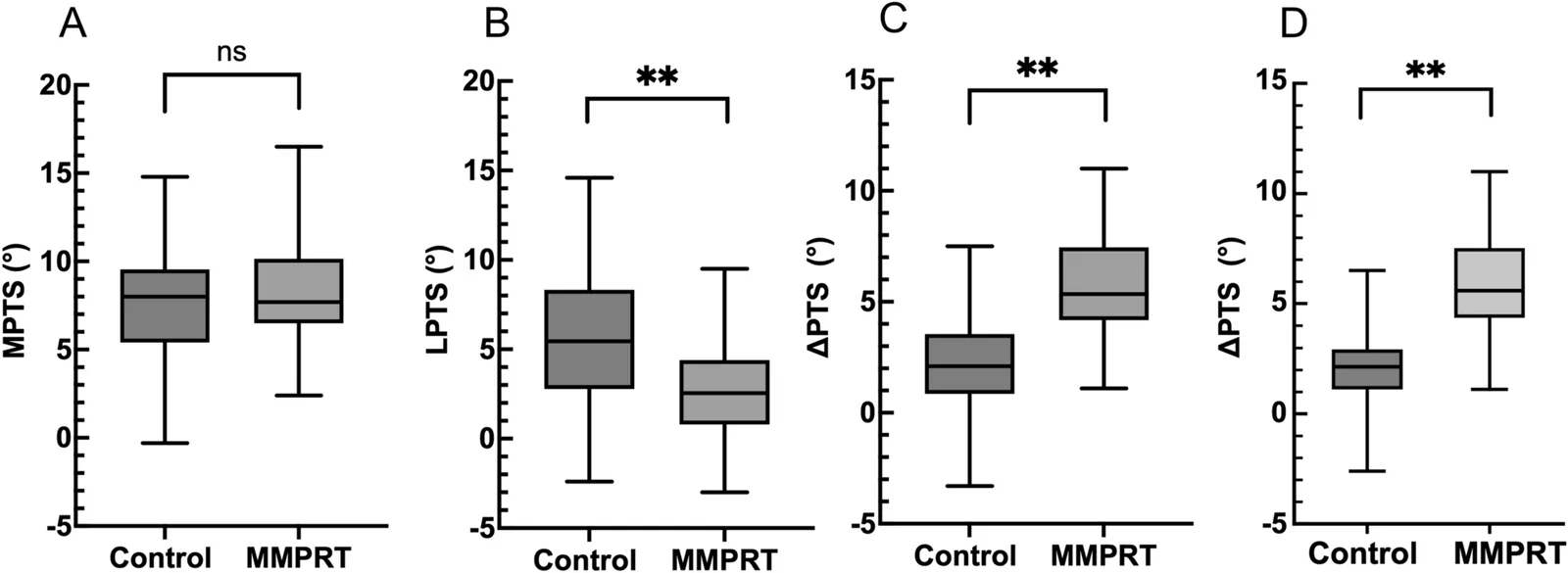
Comparison of posterior tibial slope measurements between groups before and after propensity score (PS) matching. No significant difference was observed in medial posterior tibial slope between the groups (**A**). The medial meniscus posterior root tear (MMPRT) group demonstrated a smaller lateral posterior tibial slope (**B**) and a greater ΔPTS prior to matching (**C**). After 1:1 PS matching, ΔPTS remained greater in the MMPRT group (**D**)

Table 1 summarizes the patients’ demographic characteristics and measurement data before and after PS matching. A total of 120 knees (50 males and 70 females; mean ± SD age, 59.9 ± 6.8 years) were included in the final analysis. MMPRT was observed in 26 knees (21.7%). BMI was higher in the MMPRT group (26.8 ± 4.8 versus 23.5 ± 3.4 kg/m^2^, *p* < 0.001), whereas age and sex did not differ significantly. MPTA and LDFA did not differ significantly between the groups (*p* = 0.271 and 0.810, respectively). MPTS did not differ significantly between the control and MMPRT groups (7.6 ± 2.9° versus 8.4 ± 3.3°, *p* = 0.228). In contrast, the MMPRT group showed a significantly smaller LPTS (2.7 ± 3.1° versus 5.5 ± 3.4°, *p* < 0.001) and a significantly larger ΔPTS (5.7 ± 2.4° versus 2.1 ± 2.2°, *p* < 0.001) than the control group (Fig. 3).

**Table 1:** Characteristics of patients before and after 1:1 PS matching

Covariate	Before PS matching				After PS matching			
	Control	MMPRT	*p*-value	SMD	Control	MMPRT	*p*-value	SMD
N	94	26	—	—	22	22	—	—
Age (years)	59.9 ± 7.1	60.2 ± 5.8	0.823	0.05	61.5 ± 6.2	61.2 ± 5.6	0.831	0.04
Gender (male/female)	42/52	8/18	0.203	0.29	6/16	6/16	—	0.00
Body mass index (kg/m^2^)	23.5 ± 3.4	26.8 ± 4.8	< 0.001	0.81	25.5 ± 3.1	25.5 ± 3.3	0.680	0.01
MPTA (°)	87.1 ± 1.7	87.5 ± 1.5	0.271	—	87.2 ± 1.6	87.7 ± 1.5	0.303	—
LDFA (°)	86.6 ± 1.7	86.7 ± 2.1	0.810	—	86.4 ± 1.5	86.8 ± 2.2	0.545	—
MPTS (°)	7.6 ± 2.9	8.4 ± 3.3	0.228	—	6.9 ± 2.7	8.5 ± 3.1	0.076	—
LPTS (°)	5.5 ± 3.4	2.7 ± 3.1	< 0.001	—	5.2 ± 3.4	2.7 ± 2.5	0.015	—
ΔPTS (°)	2.1 ± 2.2	5.7 ± 2.4	< 0.001	—	1.7 ± 1.9	5.8 ± 2.4	< 0.001	—

*MPTA* medial proximal tibial angle, *LDFA* lateral distal femoral angle, *MPTS* medial posterior tibial slope, *LPTS* lateral posterior tibial slope, *PS* propensity score, *SMD* standardized mean difference

Data are shown as mean ± standard deviation or number of patients. ΔPTS was calculated by MPTS − LPTS. SMDs were calculated for the matching covariates only. —, not applicable. Before matching, groups were compared using Student’s *t*-test and the *χ*^2^ test. After matching, continuous variables were compared using the paired *t*-test. *p*-values of < 0.05 were considered significant

After 1:1 PS matching, 22 matched pairs were obtained. Age, sex, and BMI were well balanced between the groups, with all SMDs below 0.1. Regarding coronal alignment, neither MPTA nor LDFA differed significantly between the groups. The MMPRT group continued to show a significantly smaller LPTS (2.7 ± 2.5° versus 5.2 ± 3.4°, *p* = 0.015) and a significantly larger ΔPTS (5.8 ± 2.4° versus 1.7 ± 1.9°, *p* < 0.001) than the control group (Fig. 3). As a sensitivity analysis, 1:2 PS matching was additionally performed in 17 matched sets, and the results were consistent with those of the main analysis (Supplementary Table 1).

Table 2 presents the multivariable models. When the slope parameters were entered separately, ΔPTS was independently associated with MMPRT (OR 2.17 per 1° increase, 95% CI 1.56–3.02, *p* < 0.001), as was LPTS (OR 0.74, 95% CI 0.62–0.88, *p* = 0.001), whereas MPTS was not (OR 1.10, 95% CI 0.94–1.29, *p* = 0.251). BMI was associated with MMPRT in every model, and neither MPTA nor LDFA were associated in any model. When MPTS and LPTS were entered simultaneously, so that each was adjusted for the other, MPTS became strongly associated with MMPRT (OR 2.07, 95% CI 1.47–2.92, *p* < 0.001) and the association of LPTS strengthened (OR 0.44, 95% CI 0.30–0.63, *p* < 0.001). The odds ratio for MPTS was close to the reciprocal of that for LPTS (1/0.44 = 2.28), indicating effects of similar magnitude in opposite directions. The model using ΔPTS as the single slope parameter and the model containing both slopes showed similar fit (AIC 77.9 and 79.1).

**Table 2:** Multivariable logistic regression models for the presence of MMPRT

Variable	Odds ratio	95% CI	*p*-value	AIC
Model 1: ΔPTS				77.9
ΔPTS (per 1°)	2.17	1.56–3.02	< 0.001	
Body mass index (per 1 kg/m^2^)	1.30	1.11–1.52	0.001	
MPTA (per 1°)	1.02	0.67–1.55	0.930	
LDFA (per 1°)	1.09	0.77–1.56	0.618	
Model 2: MPTS				118.4
MPTS (per 1°)	1.10	0.94–1.29	0.251	
Body mass index (per 1 kg/m^2^)	1.26	1.10–1.44	0.001	
MPTA (per 1°)	1.14	0.83–1.58	0.409	
LDFA (per 1°)	1.07	0.81–1.42	0.628	
Model 3: LPTS				104.6
LPTS (per 1°)	0.74	0.62–0.88	0.001	
Body mass index (per 1 kg/m^2^)	1.28	1.11–1.49	0.001	
MPTA (per 1°)	1.21	0.84–1.73	0.300	
LDFA (per 1°)	1.05	0.77–1.42	0.762	
Model 4: MPTS and LPTS				79.1
MPTS (per 1°)	2.07	1.47–2.92	< 0.001	
LPTS (per 1°)	0.44	0.30–0.63	< 0.001	
Body mass index (per 1 kg/m^2^)	1.31	1.12–1.53	0.001	
MPTA (per 1°)	1.02	0.66–1.56	0.945	
LDFA (per 1°)	1.09	0.76–1.55	0.647	

*MMPRT* medial meniscus posterior root tear, *MPTS* medial posterior tibial slope, *LPTS* lateral posterior tibial slope, *MPTA* medial proximal tibial angle, *LDFA* lateral distal femoral angle, *CI* confidence interval, *AIC* Akaike information criterion

All models were fitted in the full cohort (*n* = 120, 26 events). ΔPTS was calculated as MPTS − LPTS. Because the three slope parameters are exactly linearly dependent, they were entered in separate models. A lower AIC indicates better model fit

Institutions A and B contributed 64 and 56 knees, respectively, and the proportion with MMPRT was similar (23.4% and 19.6%, *p* = 0.615). The absolute values of MPTS and LPTS differed between institutions (*p* = 0.008 and *p* = 0.001, respectively), whereas ΔPTS did not (3.1 ± 2.9° versus 2.6 ± 2.5°, *p* = 0.260). A greater ΔPTS in the MMPRT group was observed at both institutions (2.4 ± 2.5° versus 5.4 ± 2.9° at institution A and 1.7 ± 1.8° versus 6.1 ± 1.5° at institution B, *p* < 0.001 for both), as was a smaller LPTS (*p* = 0.010 and *p* = 0.006), whereas MPTS did not differ at either institution (*p* = 0.327 and *p* = 0.342) (Supplementary Table 2). The sex distribution differed between the two institutions (*p* = 0.002), whereas age and BMI did not. In sensitivity analyses, further adjustment for age and sex (OR 2.22, 95% CI 1.56–3.17) or for institution (OR 2.17, 95% CI 1.57–3.02) left the estimate for ΔPTS unchanged, and institution itself was not associated with MMPRT (OR 1.27, 95% CI 0.33–4.83, *p* = 0.725).

**Fig. 4:**
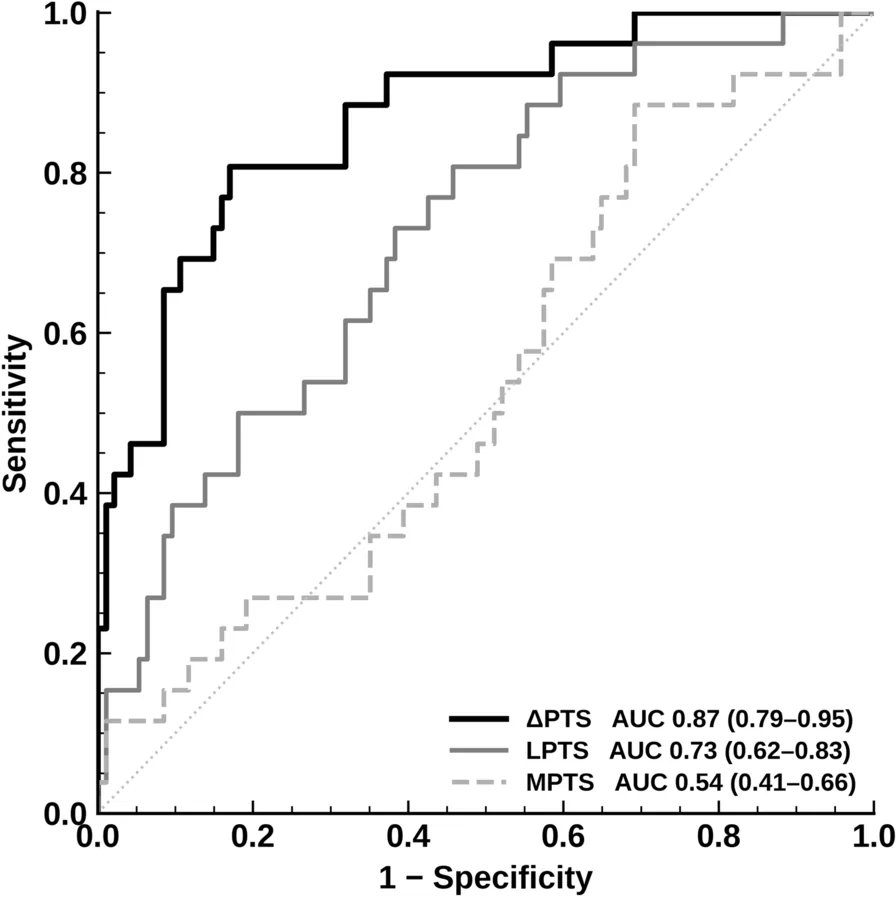
Receiver operating characteristic (ROC) curve analysis of ΔPTS, LPTS, and MPTS for identifying medial meniscus posterior root tear (MMPRT). The area under the curve was 0.87 (95% CI, 0.79–0.95) for ΔPTS, 0.73 (95% CI 0.62–0.83) for LPTS, and 0.54 (95% CI 0.41–0.66) for MPTS. ΔPTS discriminated MMPRT better than LPTS (DeLong *p* = 0.006) and better than MPTS (*p* < 0.001). The exploratory cutoff value for ΔPTS was 4.0°, yielding a sensitivity of 80.8% and a specificity of 81.9%

The AUC was 0.87 (95% CI 0.79–0.95) for ΔPTS, 0.73 (95% CI 0.62–0.83) for LPTS, and 0.54 (95% CI 0.41–0.66) for MPTS. ΔPTS discriminated MMPRT better than LPTS (*p* = 0.006) and better than MPTS (*p* < 0.001) (Fig. 4). Using the Youden index, an exploratory cutoff value of 4.0° for ΔPTS yielded a sensitivity of 80.8% and a specificity of 81.9%.

The ICC (2, 1) values were 0.77 for MPTS, 0.67 for LPTS, 0.61 for ΔPTS, 0.75 for MPTA, and 0.78 for LDFA, indicating good reliability for MPTS, MPTA, and LDFA, and moderate reliability for LPTS and ΔPTS [22]. Agreement for the MMPRT classification between the two readers was *κ* = 0.86, with concordant classification in 54 of the 57 examinations reviewed (94.7%).

## Discussion

The most important finding of this study was that ΔPTS was significantly greater in the MMPRT group than in the control group, and that this difference, rather than either slope on its own, distinguished the two groups. Before PS matching, a difference in BMI was observed between the two groups, consistent with previous reports that identify obesity as a risk factor for MMPRT [23]. Given the baseline differences in patient characteristics, PS matching was performed to adjust for potential confounding factors. After matching, the between-group comparison yielded results similar to those observed before matching, with the MMPRT group showing a significantly greater ΔPTS. This association remained independent in multivariable logistic regression after adjusting for BMI, MPTA, and LDFA, and ROC analysis confirmed that ΔPTS discriminated well between the groups.

Although medial–lateral slope asymmetry has been investigated, particularly in the context of lateral meniscus posterior root tears, there is currently limited evidence demonstrating a direct association between slope asymmetry and MMPRT [12, 13]. A cadaveric investigation has shown that increasing posterior tibial slope results in greater ATT under axial loading, particularly at low flexion angles [24]. Furthermore, increased posterior tibial slope has been suggested to promote femoral rollback and potentially contribute to impingement of the medial meniscus posterior root [9]. In the MMPRT group of the present study, LPTS was smaller relative to MPTS. Given that higher PTS is associated with increased ATT, a greater ΔPTS may lead to relatively larger ATT in the medial compartment compared with the lateral compartment. We hypothesize that this imbalance in ATT produces a rotational imbalance at the tibia, which may in turn contribute to the development of MMPRT. Herbst reported that the difference between MPTS and LPTS is associated with altered tibial rotational kinematics [11]. Furthermore, an increased PTS has been shown to increase compressive and posterior shear forces on the medial meniscus posterior root under axial loading, with tibial rotational torque further altering root shear [25]. This asymmetry would therefore concentrate axial loading in the medial compartment, elevating mechanical stress on the medial meniscus posterior root.

Although previous studies have suggested that increased MPTS is associated with a higher risk of MMPRT [7, 8], MPTS did not differ significantly between groups in this study. Three factors may contribute to this discrepancy. First, with 26 knees in the MMPRT group, the power to detect a difference in MPTS may have been limited. Second, the control group consisted not of healthy knees but of symptomatic knees that underwent MRI for knee pain and had no MMPRT. Because a steep medial slope has been associated with degenerative medial meniscal pathology in general [8], the control group may itself have been enriched for steeper medial slopes, which would narrow the difference between the groups. Third, absolute PTS values are sensitive to how the tibial reference axis is defined. Lipps demonstrated that differences in the definition of the tibial proximal anatomical axis, including variations in the selected tibial length used for axis construction, may result in differences in measured slope values [26]. Absolute MPTS values are therefore not directly comparable across studies that use different imaging modalities or axis definitions. Importantly, however, ΔPTS is a relative parameter derived from medial and lateral measurements obtained on the same reference axis, so systematic errors related to axis definition are likely to be canceled. The present multicenter data support this, as the absolute values of MPTS and LPTS differed between the two institutions whereas ΔPTS did not.

Because the between-group difference in ΔPTS was accompanied by a smaller LPTS, we also examined whether ΔPTS conveys information beyond LPTS alone. ΔPTS discriminated MMPRT better than LPTS (area under the curve 0.87 versus 0.73, *p* = 0.006), and MPTS, which showed no association on its own, became strongly associated with MMPRT once LPTS was included in the same model. In that model, a higher MPTS at a fixed LPTS means a larger difference between the two slopes, hence, the model detects the asymmetry rather than the absolute medial slope. These findings indicate that sagittal plane asymmetry, rather than the absolute value of either slope, is more relevant to MMPRT pathomechanics. The medial slope is therefore relevant to MMPRT, but its relevance becomes apparent only when it is considered relative to the lateral slope, which reconciles the present findings with previous reports.

Beyond outperforming either slope alone, the AUC of 0.87 for ΔPTS represents a level of discrimination generally considered clinically meaningful [27]. The balanced sensitivity (80.8%) and specificity (81.9%) suggest that ΔPTS provides stable classification performance. Notably, the cutoff value was relatively small (4.0°), indicating that even subtle medial–lateral slope differences may be clinically relevant. ΔPTS may therefore serve as an adjunctive parameter alongside conventional MRI assessment rather than a standalone diagnostic criterion. Because ΔPTS and MMPRT status were assessed on the same MRI examination, the present analysis describes discrimination between knees with and without an existing tear rather than prediction of a future tear. The 4.0° cutoff was derived from a single relatively small cohort without external validation and should therefore be regarded as an exploratory threshold rather than an established screening criterion.

Several risk factors for MMPRT have been previously reported, including age over 50 years, female sex, obesity, varus alignment, and low activity level [23]. Given that varus alignment may vary by sex and race, these factors may have partly influenced the present findings [28]. In addition to bony morphology, factors that alter tibial rotational loading may also be relevant to MMPRT. Abnormal proximal mechanics, including excessive femoral internal rotation and hip adduction, have been shown to increase dynamic knee valgus and rotational malalignment [29], and patients with MMPRT risk profiles may frequently present with such functional abnormalities. These observations suggest that the present findings may have implications for future preventive strategies targeting modifiable biomechanical factors associated with MMPRT.

This study has several limitations. First, the number of knees with MMPRT was relatively small (*n* = 26), which limits statistical power. Even the significant findings require confirmation in larger cohorts. Second, the proposed mechanism of rotational imbalance was not directly evaluated, because the study did not include dynamic biomechanical assessment. Motion analysis or in vivo kinematic studies are required to test this hypothesis. Third, factors known to influence MMPRT such as activity level and meniscal extrusion could not be evaluated in this retrospective cohort, and coronal alignment was estimated from MRI rather than from full-length standing radiographs. Because MRI-based MPTA and LDFA have not been validated against that standard, residual confounding cannot be excluded. The interobserver reliability of ΔPTS was also only moderate, although such measurement error would weaken rather than create the observed association. Fourth, symptom duration was not recorded, so acute-onset and chronic tears could not be distinguished. Whether the association between ΔPTS and MMPRT differs by chronicity remains to be clarified. Fifth, patients with advanced osteoarthritis (Kellgren–Lawrence grade 3 or 4) were excluded, so the findings may not be generalizable to advanced degenerative knees, in which MMPRT also frequently occurs. Sixth, although patients were screened consecutively from the imaging records of both institutions, the number excluded by each criterion was not recorded, so a detailed flow of patient selection cannot be presented.

## Conclusions

Knees with MMPRT exhibited a smaller LPTS and a greater ΔPTS than knees without MMPRT, and these differences remained significant after PS matching and multivariable adjustment. ROC analysis demonstrated good discriminative ability, identifying a ΔPTS of 4.0° as an exploratory threshold. These findings suggest that medial–lateral PTS asymmetry may be a clinically relevant morphological factor associated with MMPRT. Further large-scale and biomechanical studies are warranted to validate this threshold and clarify the underlying mechanism.

## Supplementary Information

Supplementary Material 1.

Supplementary Material 2.

## Data Availability

The data that support the findings of this study are available from the corresponding author upon reasonable request. Due to ethical restrictions and patient confidentiality, the data are not publicly available.

## Abbreviations

MMPRT: Medial meniscus posterior root tear
BMI: Body mass index
PTS: Posterior tibial slope
ATT: Anterior tibial translation
MPTA: Medial proximal tibial angle
LDFA: Lateral distal femoral angle
PACS: Picture archiving and communication system
ICC: Intraclass correlation coefficient
MPTS: Medial posterior tibial slope
LPTS: Lateral posterior tibial slope
PS: Propensity score
SMD: Standardized mean difference
AIC: Akaike information criterion
OR: Odds ratio
CI: Confidence interval
ROC: Receiver operating characteristic
AUC: Area under the curve
SD: Standard deviation
